# Dietary Patterns and Depressive Symptom Severity in the Hungarian Adult Population: Evidence from a Nationally Representative Survey

**DOI:** 10.3390/nu18010159

**Published:** 2026-01-03

**Authors:** Battamir Ulambayar, Attila Csaba Nagy

**Affiliations:** Department of Epidemiology, Faculty of Health Sciences, University of Debrecen, 4032 Debrecen, Hungary; ulambayar.battamir@etk.unideb.hu

**Keywords:** dietary patterns, depressive symptoms, PHQ-8, European Health Interview Survey, Hungary

## Abstract

***Background:*** Depression represents a major public health burden in Hungary, where prevalence remains higher than the global average. Although diet is an increasingly studied factor associated with mental health, evidence from Central and Eastern Europe is scarce. ***Methods:*** This cross-sectional study analyzed data from the Hungarian wave of the European Health Interview Survey (EHIS) 2019, a nationally representative sample of 5603 adults aged ≥15 years. Depressive symptom severity was assessed using the Patient Health Questionnaire-8 (PHQ-8) and categorized as none (0–4), mild (5–9), and moderate-to-severe (≥10). Self-reported frequency of consumption of fruits, vegetables, fruit juice, dairy products, fish, processed meat, sweetened beverages, coffee, and sweeteners was examined. Multivariable ordinal logistic regression models, adjusted for gender, age, education, income, physical activity, smoking, and alcohol consumption, were used to estimate associations with depressive symptom severity. ***Results:*** Overall, 77.9% of participants had no depression, 17.0% mild, and 5.1% moderate-to-severe symptoms. After full adjustment, lower consumption of fruits and vegetables, less frequent fruit juice intake, and lower processed meat consumption were associated with higher odds of more severe depressive symptoms. Moderate coffee intake (1–2 cups/day) was associated with lower odds than heavier consumption. ***Conclusions:*** In the Hungarian adult population, poorer dietary patterns, particularly low intake of fruits, vegetables, and paradoxically lower processed meat consumption, are significantly associated with greater depressive symptom severity, independent of major sociodemographic and lifestyle factors. These findings underscore the potential role of diet quality in mental health and support public health efforts to promote nutrient-rich dietary patterns in Hungary.

## 1. Introduction

Depression is a major public health challenge, and its prevalence is increasing predominantly [1]. Its impact extends across all age groups, contributing substantially to reduced quality of life, increased healthcare utilization, and premature mortality [2]. In Europe, and particularly in Central and Eastern European countries, the prevalence of depressive symptoms is higher compared to the worldwide [3,4], underscoring the need to better understand modifiable determinants that may influence mental health outcomes. Hungary is no exception, with persistently high levels of depressive symptoms and suicide rates associated with depression [5]. Identifying risk and protective factors within this context is essential for developing effective prevention and intervention strategies.

In recent years, growing attention has been directed toward the role of lifestyle factors, especially diet, in shaping mental health [6]. A growing body of evidence suggests that low-inflammatory diet patterns rich in fruits, vegetables, whole grains, fish, and other nutrient-dense foods may be protective against depression, while diets high in processed foods, added sugars, and saturated fats may increase risk [7]. Potential mechanisms include nutritional influences on inflammation, oxidative stress, neurotransmitter synthesis, and the gut–brain axis [8]. Although these associations have been explored in several populations, evidence remains inconsistent, and relatively few studies have examined them in Central or Eastern Europe, where dietary habits, food environments, and socioeconomic conditions differ from those in Western European countries.

Hungary presents a unique setting to assess the associations between dietary patterns and depression because it has traditional dietary patterns, marked by high consumption of processed meats, refined grains, and low intake of fresh produce [9]. These dietary habits are further influenced by cultural norms, regional food availability, and socioeconomic factors, which may contribute to nutritional imbalances and increased risk of mental health disorders. Moreover, Hungary has experienced rapid dietary transitions over recent decades, with greater exposure to Western-style diets and ultra-processed foods, making it an ideal context to explore how both traditional and modern eating habits intersect with psychological well-being [10]. At the same time, changing food environments, increasing health awareness, and socioeconomic gradients in dietary quality highlight the importance of understanding how eating habits relate to mental well-being at the population level [11]. Despite these considerations, evidence on the relationship between dietary patterns and depressive symptoms in Hungarian adults remains limited.

To address this gap, the present study uses nationally representative data to examine the association between dietary behaviors and depressive symptom severity in the Hungarian adult population. By evaluating a wide range of eating habits, including fruit and vegetable intake, consumption of dairy products, fish, meat, sweetened beverages, coffee, and sweeteners, we aim to provide a comprehensive understanding of how everyday dietary choices relate to depression. We hypothesized that lower consumption of nutrient-rich foods would be associated with greater depressive symptom severity, whereas more favorable dietary behaviors would be associated with lower severity.

## 2. Materials and Methods

### 2.1. Study Design and Data Source

This cross-sectional study utilized data from the European Health Interview Survey (EHIS) 2019, the most recent wave conducted in Hungary. The EHIS is a standardized, population-based survey coordinated by Eurostat to collect harmonized health-related information across European Union Member States. The Hungarian Central Statistical Office (HCSO) carried out data collection in 2019 using a multistage, stratified sampling design to obtain a nationally representative sample of the non-institutionalized adult population aged 15 years and older [12]. Sampling is stratified by geographic region and settlement characteristics, with households selected at the first stage and one eligible individual randomly selected within each household. Survey weights provided by Eurostat were applied in all analyses to account for unequal selection probabilities, non-response, and to ensure representativeness of the Hungarian adult population. Data were collected through personal interviews and self-administered questionnaires. The survey included modules on sociodemographic characteristics, health status, lifestyle and dietary habits, mental health, and healthcare use. The study population consisted of individuals aged 15 years and older who participated in the Hungarian wave of the EHIS 2019. Participants were eligible for inclusion if they were members of the non-institutionalized population, completed the PHQ-8 questionnaire, and provided valid responses for the dietary exposure of interest. Individuals were excluded if they had missing data on depressive symptom severity, key dietary variables, or essential covariates used in the multivariable analyses.

### 2.2. Variables

The primary outcome was depressive symptom severity, measured using the Patient Health Questionnaire-8 (PHQ-8) [13]. Total scores were categorized according to established cut-offs: no depression: 0–4, mild depression: 5–9, moderate or severe depression: ≥10 [14]. This three-level ordered variable was used in all analyses.

Dietary habits were assessed using the standardized dietary module of the EHIS, developed and coordinated by Eurostat and implemented uniformly across European Union Member States. It includes predefined items assessing the usual frequency of consumption of selected food groups and beverages, such as fruits, vegetables, fruit juice, dairy products, fish, meat, sweetened beverages, coffee, sweets, salt, and sweeteners used for hot drinks. Data were collected via self-administered questionnaires following the face-to-face interview, in accordance with EHIS methodological guidelines [12].

Demographic, socioeconomic, and lifestyle factors known to influence mental health were used as covariates, including gender, age group, education level, household income quintiles, characteristics of main daily physical activity, smoking status, and alcohol consumption.

### 2.3. Statistical Analysis

Descriptive statistics were used to summarize sample characteristics. Categorical variables were presented as frequencies and weighted percentages. Bivariate associations between depressive symptom categories and covariates were examined using the chi-square test. We used multivariable ordinal logistic regression to examine the independent relationships between dietary habits and the severity of depressive symptoms. Variables that were significantly associated with depressive symptom severity in bivariate analyses were retained in the final multivariable ordinal logistic regression model. To confirm the appropriateness of the ordinal logistic regression model, we tested the proportional odds (parallel lines) assumption with the Wald test. No significant violations were detected (*p* > 0.05), indicating that the proportional odds model was suitable for our analysis. The model covariates included demographic, socioeconomic, lifestyle, and dietary variables. Adjusted odds ratios (ORs) with 95% confidence intervals (CIs) were reported. Survey weights were applied in all analyses to account for the complex sampling design. All statistical analyses were performed using STATA IC version 18.0 (StataCorp LLC, College Station, TX, USA) [15], with significance set at *p* < 0.05.

### 2.4. Ethical Considerations

This study involved secondary analysis of anonymized data from the EHIS 2019. The studies involving human participants were reviewed and approved by the Ethics Committee of the University of Debrecen (approval number: 5609-2020, dated 17 December 2020). The research was conducted in accordance with local legislation and institutional requirements. Written informed consent for participation was not required from the participants or their legal guardians/next of kin, in accordance with national legislation and institutional requirements.

## 3. Results

The study included 5603 participants, of whom 54.1% were female. Nearly half were aged 35–64 years (48.2%), with 22.8% aged 15–34 and 29.1% aged ≥65. Most had completed secondary education (56.2%), followed by higher (22.3%) and primary education (21.5%). Household income was relatively balanced across quintiles, ranging from 15.5% in the highest to 22.7% in the fourth quintile. Regarding physical activity, 41.6% reported moderate exertion, 36.1% were mostly sedentary, 10.8% engaged in light activity, 6.2% performed heavy work, and 3.7% had no regular activity. Over half were never smokers (53.6%), while 26.4% were current and 18.8% former smokers. Most participants reported low or moderate alcohol consumption (63.4%), 30.3% were non-drinkers, and 5.1% engaged in high-risk drinking (Table 1).

Based on PHQ-8 scores, 4365 individuals (77.9%) were classified as having no depression, 953 participants (17.0%) had mild depressive symptoms, and 285 participants (5.1%) were classified as having moderate or severe depression.

Table 2 presents the distribution of depressive symptom severity across demographic, socioeconomic, and lifestyle factors in the Hungarian adult population. Women reported significantly higher levels of depressive symptoms than men. While 82.1% of men had no depression, only 74.4% of women fell into this category, and women showed higher proportions of mild and moderate-to-severe depression (*p* < 0.001). Depression severity varied substantially by age group. Younger adults (15–34 years) had the lowest prevalence of moderate-to-severe depression (4.3%), whereas adults aged 65 and older showed the highest (7.6%). Similarly, mild depression increased with age (*p* < 0.001). A strong inverse association was observed between education and depression severity. Individuals with primary education had the highest prevalence of moderate-to-severe symptoms (10.1%), compared with only 2.4% among those with higher education (*p* < 0.001). Depression severity decreased with increasing income. The lowest income quintile exhibited the highest proportion of moderate-to-severe depression (8.9%), whereas the highest income group had the lowest (2.7%). This gradient was statistically significant (*p* < 0.001).

In terms of lifestyle factors, respondents who were mostly sedentary had higher levels of depression than those engaged in light, moderate, or heavy physical activity. Notably, individuals not performing work or regular activity had the highest proportion of moderate-to-severe depression (17.7%) (*p* < 0.001). High-risk alcohol users showed a higher prevalence of mild and moderate-to-severe depression compared with moderate and non-drinkers. Non-drinkers also exhibited elevated rates of depression compared to moderate drinkers, indicating a J-shaped association (*p* < 0.001).

Table 3 shows the associations between various dietary habits and the severity of depressive symptoms. Lower consumption of fruits and vegetables was strongly associated with higher depression severity. Individuals consuming fruits or vegetables less than once a week had approximately double the proportion of moderate-to-severe depression compared with daily consumers (*p* < 0.001). Participants who consumed fruit juice less than once a week showed higher rates of both mild and moderate-to-severe depression compared with daily consumers (*p* < 0.001). Coffee consumption frequency demonstrated a modest association with depression severity (*p* = 0.008). Individuals consuming coffee 1–2 times per day had lower proportions of moderate-to-severe depression (4.1%) compared with both heavy consumers (>3/day) and infrequent consumers (<1/day). Artificial sweetener users showed slightly higher proportions of mild and moderate-to-severe depression (19.5% and 6.0%, respectively) compared with natural sweetener users and those using no sweetener (*p* = 0.015). For the meat product consumptions, processed meat consumption showed a clear gradient, lower consumption frequency was associated with higher depression severity (*p* < 0.001). Lower dairy consumption was associated with higher depression severity (*p* = 0.003). Participants consuming dairy less than once a week had the highest proportion of moderate-to-severe symptoms (7.4%).

The results of the multivariable ordered logistic regression assessing factors associated with increasing severity of depressive symptoms are shown in Table 4. Women had significantly higher odds of more severe depressive symptoms than men (OR = 1.54; 95% CI: 1.31–1.80). Older adults (≥65 years) had markedly higher odds of more severe depression compared with those aged 18–34 years (OR = 1.86). Compared with those with only primary education, individuals with secondary (OR = 0.61) and higher education (OR = 0.54) had substantially lower odds of more severe depressive symptoms. A consistent gradient was observed, with higher income groups showing progressively lower odds of depression severity. The highest income quintile had the lowest risk (OR = 0.52), indicating strong socioeconomic protection. Compared to individuals with mostly sedentary activity, those engaging in light, moderate, or heavy physical activity had significantly lower odds of more severe depressive symptoms. In contrast, individuals not performing any regular work or activity had increased odds of depression (OR = 1.53). The moderate alcohol consumption was associated with reduced odds of depression severity (OR = 0.59), as was non-drinking (OR = 0.64), compared with high-risk drinkers.

After adjusting for demographic, socioeconomic, and lifestyle variables, several significant dietary predictors of depression severity remained. Lower frequency of fruit and vegetable consumption independently predicted higher depressive symptom severity. Individuals consuming fruit less than once a week (OR = 1.33) and those consuming vegetables less than once a week (OR = 1.53) had significantly increased odds of more severe depression. Both moderate (once or more a week) and low (<1/week) fruit juice intake were associated with higher odds of depression compared with daily consumption (ORs 1.27 and 1.30, respectively). Consuming coffee 1–2 times per day was associated with lower odds of depression severity compared with heavy coffee consumption (OR = 0.76). Lower processed meat consumption was significantly associated with higher depression severity. Compared with individuals consuming processed meat 4–7 times a week, those consuming it 1–3 times a week (OR = 1.21) or less than once a week (OR = 1.53) had higher odds of experiencing more severe depressive symptoms.

## 4. Discussion

The primary objective of this study was to investigate the association between habitual dietary patterns and the severity of depressive symptoms in a nationally representative sample of the Hungarian population. Our findings indicate that several common dietary behaviors are independently associated with the severity of depressive symptoms, even after adjusting for sex, age, education, income, physical activity, smoking, and alcohol consumption. These results are discussed in detail below.

Age has been recognized as a contributing factor to depression risk, but its relationship with depression is complex. The association between older age and depression is noted; however, it is often confounded with other variables like education and marital status. Some research specifies that older adults may experience depressive symptoms as a consequence of physical health issues or lack of social support, particularly if they are single or have lower educational backgrounds [16]. Gender differences also play a crucial role in the prevalence of depression. Findings consistently show that women are more frequently diagnosed with depression compared to men, potentially due to biological, social, and psychological factors [17,18]. Educational attainment is another significant predictor; higher education levels correlate with lower depression severity. Individuals with lower educational backgrounds are at a heightened risk for depression, likely due to economic insecurities and limited access to resources, which has been evidenced in various studies [19]. Socioeconomic status, particularly income, further exacerbates this risk, individuals from lower income brackets are consistently found to have higher rates of depression [20]. These are completely aligned with our results, which demonstrate clear associations between demographic and socioeconomic variables and the severity of depression in the Hungarian population.

Beyond these sociodemographic determinants, lifestyle behaviors represent additional modifiable factors that may influence depressive symptom severity. In our results, physical activity and alcohol consumption were associated with depression as lifestyle factors. Physical activity has consistently shown a beneficial effect on mental health, including reducing the severity of depressive symptoms. Engaging in regular exercise is linked to improved mood and decreased anxiety and depression levels [21,22]. This relationship is partially attributed to biological mechanisms such as the enhancement of neurotrophic factors and regulation of neurotransmitters that are critical in mood stabilization [23]. Alcohol consumption presents a more complex relationship with depression. Moderate alcohol intake has been debated in the context of its potential protective effects against depression, particularly among those who also engage in other healthy lifestyle behaviors [24]. However, increased severity of depressive symptoms has been correlated with heavier alcohol consumption, especially when misuse occurs. A systematic review also reinforced that heavy drinking poses a considerable risk for developing depressive disorders, marking a clear distinction from light to moderate consumption, which could be less harmful under certain conditions [25]. While lifestyle behaviors such as physical activity and alcohol consumption play an important role, dietary habits constituted the primary focus of the present study and are discussed in detail below.

Regarding dietary patterns, the main focus of the current study, lower fruit juice intake, lower fruit and vegetable consumption, were associated with the severity of depression independently, in our results. The relationship between fruit and vegetable consumption and depression severity has garnered increasing interest in recent years, with compelling evidence suggesting a negative association between fruit and vegetable intake and depressive symptoms, consistent with our findings. For example, a study based on the 2014 Korea National Health and Nutrition Examination Survey found that decreased consumption of fruits and vegetables was linked with higher levels of depression and psychological distress among adults [26]. Furthermore, a meta-analysis involving over 200,000 participants confirmed that higher intake of fruits and vegetables is inversely associated with the risk of depression, reinforcing the protective role of these foods against mental health disorders [27]. In the Iranian population, consumption patterns that include a higher intake of fruits, particularly dried fruits and juices, were found to be inversely associated with severe depression, anxiety, and stress [28]. Biologically, fruits are rich in essential nutrients such as vitamins, antioxidants, and dietary fiber, all of which are believed to play a role in mental health. Folate, prevalent in many fruits, is particularly notable, as its deficiency has been directly linked to depressive symptoms [29]. Additionally, carotenoids, found abundantly in colorful fruits, may protect against oxidative stress, which is associated with mood disorders [30]. Beyond the biochemical interactions, lifestyle factors also intersect with dietary intake. A study indicated that low fruit and vegetable consumption was prevalent among individuals with low social support, an established risk factor for depression, suggesting that dietary habits may be influenced by social networks and overall well-being [31].

In addition to these plant-based foods, beverage consumption patterns, particularly coffee intake, also demonstrated noteworthy associations with depressive symptoms. The relationship between coffee consumption and the severity of depression has been examined extensively, showing a generally inverse association. This suggests that higher coffee consumption is often linked to a lower prevalence or severity of depressive symptoms. One major finding is that coffee appears to have a protective effect against depression. Nouri-Majd et al. indicated that coffee intake may reduce the risk of depression, noting that certain studies support a relationship where coffee consumption is associated with a lower prevalence of depressive symptoms, unlike caffeine alone [32]. This is supported in meta-analyses and systematic reviews that demonstrate an overall trend, suggesting that higher coffee consumption correlates with decreased depression severity [33,34]. Additionally, Lucas found a nuanced dose–response relationship, where moderate coffee consumption was associated with a reduced risk of depression, but excess consumption was linked to adverse outcomes, potentially due to individuals using high doses for self-medication without effect [35]. This observation indicates the complexity of the relationship, suggesting that while moderate consumption may confer benefits, similar to our results, but excessive intake could be unfavorable.

An unexpected but robust finding of the present study was that a lower frequency of processed meat consumption was independently associated with higher odds of more severe depressive symptoms after full adjustment. This direction contrasts with most observational evidence from Western populations, where higher intake of processed meat is consistently linked to increased depression risk through pro-inflammatory mechanisms and poor overall diet quality [36]. In the Hungarian context, however, frequent consumption of processed meats such as salami, sausages, and smoked products remains an integral part of the traditional dietary pattern and is often consumed within balanced home-cooked meals that also include vegetables, dairy, and soups [9]. Consequently, deliberately reducing processed meat intake may reflect health-related dietary modifications prompted by existing physical or mental illness, physician advice, or early symptoms of depression itself, introducing reverse causation [37,38]. Loss of appetite and anhedonia, core features of depression [39], may further decrease the desire for strongly flavoured traditional foods, while the EHIS questionnaire does not capture overall ultra-processed food intake, potentially misclassifying modern unhealthy diets as “low processed meat”.

This study has several strengths. It draws on data from a large, nationally representative sample of Hungarian adults, enhancing the generalizability of the findings. The use of standardized instruments, including the validated PHQ-8 and harmonized EHIS dietary and lifestyle measures, ensures methodological consistency and facilitates comparison with international studies. The comprehensive adjustment for sociodemographic and lifestyle covariates, along with the simultaneous examination of multiple dietary behaviors, provides a robust assessment of independent associations between diet and depressive symptom severity.

However, the limitations should also be acknowledged. the cross-sectional design precludes any inference of causal relationships between dietary behaviors and depressive symptom severity. The observed associations may be bidirectional, and reverse causation is possible, particularly if existing depressive symptoms influenced dietary choices. Dietary intake was assessed using self-reported frequency-based questions, which are subject to recall bias, reporting errors, and potential underreporting. Moreover, the EHIS dietary module captures consumption frequency rather than quantitative intake, and therefore does not provide information on actual amounts of foods consumed, nutrient composition, or total energy intake. This limitation may have led to exposure misclassification and restricts more detailed nutritional interpretation. Although we adjusted for a broad range of sociodemographic and lifestyle factors, residual confounding cannot be ruled out. Unmeasured variables such as chronic medical conditions, medication use, psychological stress, social support, or other health-related behaviors may have influenced depressive symptom severity and contributed to the observed associations. The inverse association observed between lower processed meat consumption and greater depressive symptom severity is unexpected and should be interpreted with caution. This paradoxical finding may reflect unmeasured confounding, cultural dietary patterns specific to Hungary, health-related behavioral changes, or reverse causation rather than a true protective effect. Taken together, these limitations highlight the need for longitudinal studies and intervention-based research to better clarify the directionality and underlying mechanisms linking dietary patterns and depression.

## 5. Conclusions

This nationally representative study demonstrates that dietary patterns are significantly associated with depressive symptom severity among Hungarian adults. Lower consumption of fruits, vegetables, fruit juice, and processed meat was consistently linked with higher levels of depressive symptoms, even after adjusting for key demographic, socioeconomic, and lifestyle factors. These findings suggest that dietary quality may play an important role in mental well-being, with healthier eating habits potentially offering protection against more severe depressive symptoms.

The results highlight the need for public health initiatives that promote balanced dietary patterns as part of broader mental health strategies. Encouraging regular consumption of nutrient-rich foods and improving access to healthier dietary options may contribute to reducing the burden of depression in the Hungarian population. Further research is warranted to explore causal pathways and to evaluate whether dietary interventions could effectively mitigate depressive symptoms.

## Figures and Tables

**Table 1:** Sociodemographic and lifestyle characteristics of the study population.

Variable	Category	*n*	%
Gender	Male	2572	45.9
	Female	3031	54.1
Age group	15–34 years	1276	22.8
	35–64 years	2699	48.2
	≥65 years	1628	29.1
Education level	Primary	1204	21.5
	Secondary	3147	56.2
	Higher	1252	22.3
Household income quintile	First (lowest)	1153	20.6
	Second	1173	20.9
	Third	1139	20.3
	Fourth	1269	22.7
	Fifth (highest)	869	15.5
Physical effort at work/main activity	Mostly walking/light effort	603	10.8
	Heavy, strenuous physical work	345	6.2
	Moderate physical exertion	2331	41.6
	Mostly sitting	2025	36.1
	No regular work/activity	209	3.7
Smoking status	Current smoker	1478	26.4
	Former smoker	1053	18.8
	Never smoker	3005	53.6
Alcohol consumption	High-risk drinking	284	5.1
	Low or moderate	3555	63.4
	Non-drinker	1697	30.3

**Table 2:** Associations between demographic, socioeconomic status, lifestyle factors, and the severity of depression.

Variables	Categories	Severity of Depression (PHQ-8)			*p* Value *
		No Depression	Mild Depression	Moderate or Severe Depression	
Gender	Male	2111 (82.1)	363 (14.1)	98 (3.8)	**<0.001**
	Female	2254 (74.4)	590 (19.4)	187 (6.2)	
Age group	15–34 years old	1022 (80.1)	199 (15.6)	55 (4.3)	**<0.001**
	55–64 years old	2196 (81.4)	397 (14.7)	106 (3.9)	
	65 and older	1147 (70.5)	357 (21.9)	124 (7.6)	
Education levels	Primary	796 (66.1)	287 (23.8)	121 (10.1)	**<0.001**
	Secondary	2530 (80.4)	483 (15.4)	134 (4.2)	
	High	1039 (83.0)	183 (14.6)	30 (2.4)	
Quintiles based on net equivalent household income	First (lowest)	805 (69.8)	246 (21.3)	102 (8.9)	**<0.001**
	Second	910 (77.6)	197 (16.8)	66 (5.6)	
	Third	877 (77.0)	205 (18.0)	57 (5.0)	
	Fourth	1035 (81.6)	197 (15.5)	37 (2.9)	
	Fifth (highest)	738 (84.9)	108 (12.4)	23 (2.7)	
Physical effort at work/main activity	Mostly consists of walking/light effort	493 (81.8)	93 (15.4)	17 (2.8)	**<0.001**
	Does mostly heavy, strenuous physical work	284 (82.3)	50 (14.5)	11 (3.9)	
	Mostly walks or does moderate physical exertion	1863 (79.9)	366 (15.7)	102 (4.4)	
	Mostly sitting	1525 (75.3)	385 (19.0)	115 (5.7)	
	Does not perform such activities or work of any kind	121 (57.9)	51 (24.4)	37 (17.7)	
Smoking status	Active smoker	1141 (77.2)	253 (17.1)	84 (5.7)	0.570
	Former smoker	820 (77.9)	175 (16.6)	58 (5.5)	
	Never smoker	2353 (78.3)	513 (17.1)	139 (4.6)	
Alcohol consumption	High risk	211 (74.3)	58 (20.4)	15 (5.3)	**<0.001**
	Low or moderate	2860 (80.5)	560 (15.7)	135 (3.8)	
	No alcohol use	1240 (73.1)	325 (19.2)	132 (7.8)	

* Bold values indicate statistical significance (*p* < 0.05) based on Pearson’s chi-squared test.

**Table 3:** Associations between eating habits and the severity of depression.

Variables	Categories	Severity of Depression (PHQ-8)			*p* Value *
		No Depression	Mild Depression	Moderate or Severe Depression	
Fruit consumption	Everyday	2516 (79.4)	506 (16.0)	145 (4.6)	**<0.001**
	Once or more a week	1454 (76.7)	350 (18.5)	92 (4.8)	
	Less than once a week	351 (71.3)	94 (19.1)	47 (9.6)	
Vegetable consumption	Everyday	2034 (81.1)	373 (14.9)	100 (4.0)	**<0.001**
	Once or more a week	1931 (75.8)	478 (18.6)	140 (5.6)	
	Less than once a week	347 (70.8)	99 (20.2)	44 (9.0)	
Fruit juice consumption	Everyday	385 (86.1)	44 (9.9)	18 (4.0)	**<0.001**
	Once or more a week	1112 (81.3)	209 (15.3)	47 (3.4)	
	Less than once a week	2792 (75.4)	692 (18.7)	218 (5.9)	
Sugary soft drinks	Everyday	487 (77.8)	103 (16.5)	36 (5.7)	0.441
	Once or more a week	1017 (79.5)	202 (15.8)	60 (4.7)	
	Less than once a week	2804 (77.2)	642 (17.7)	188 (5.2)	
Coffee consumption	3 or more a day	1052 (76.5)	249 (18.1)	74 (5.4)	**0.008**
	1–2 times a day	2818 (79.3)	585 (16.6)	147 (4.1)	
	Less than once a day	480 (74.8)	119 (18.5)	43 (6.7)	
Sweetener for hot drinks	Natural sweetener	2436 (78.8)	502 (16.2)	153 (5.0)	**0.015**
	Artificial sweetener	683 (74.5)	179 (19.5)	55 (6.0)	
	No sweetener	721 (79.8)	150 (16.6)	32 (3.6)	
Sweets and desserts a day	More than 3 portions	244 (73.3)	70 (21.0)	19 (5.7)	0.257
	1–2 portions	1927 (77.8)	430 (17.3)	121 (4.9)	
	Less than one portion	2150 (78.4)	451 (16.4)	143 (5.2)	
Red meat consumption	4–7 times a week	544 (78.8)	107 (15.5)	39 (5.7)	0.134
	1–3 times a week	2620 (78.6)	558 (16.7)	156 (4.7)	
	Less than once a week	1148 (75.7)	282 (18.6)	86 (5.7)	
White meat consumption	4–7 times a week	1014 (79.0)	203 (15.8)	66 (5.2)	0.213
	1–3 times a week	3106 (77.7)	695 (17.4)	197 (4.9)	
	Less than once a week	207 (74.2)	51 (18.3)	21 (7.5)	
Processed meat consumption	4–7 times a week	2308 (81.0)	409 (14.4)	132 (4.6)	**<0.001**
	1–3 times a week	1600 (75.3)	425 (20.0)	100 (4.7)	
	Less than once a week	420 (71.6)	115 (19.6)	52 (8.8)	
Fish consumption	4–7 times a week	92 (80.0)	19 (16.5)	4 (3.5)	**<0.001**
	1–3 times a week	1157 (83.2)	184 (13.2)	49 (3.6)	
	Less than once a week	3063 (75.9)	744 (18.4)	228 (5.7)	
Dairy product consumption	4–7 times a week	2798 (79.2)	574 (16.3)	159 (4.5)	**0.003**
	1–3 times a week	1101 (76.0)	266 (18.4)	81 (5.6)	
	Less than once a week	427 (73.5)	111 (19.1)	43 (7.4)	
Salt consumption	Low	3040 (78.4)	635 (16.8)	187 (4.8)	0.362
	Moderate	1056 (76.3)	250 (18.0)	79 (5.7)	
	High	221 (78.1)	44 (15.6)	18 (6.3)	

* Bold values indicate statistical significance (*p* < 0.05) based on Pearson’s chi-squared test.

**Table 4:** Multivariable ordered logistic regression of depressive symptom severity.

Variables	Categories	OR	95% CI	*p*-Value
Gender	Male (Ref)			
	Female	1.54	1.31–1.80	**<0.001**
Age group	18–34 years old (Ref)			
	35–64 years old	1.14	0.93–1.41	0.218
	65 years and older	1.86	1.47–2.34	**<0.001**
Education	Primary (Ref)			
	Secondary	0.61	0.51–0.72	**<0.001**
	Higher	0.54	0.41–0.69	**<0.001**
Household income	First quintile (Ref)			
	Second quintile	0.64	0.52–0.80	**<0.001**
	Third quintile	0.78	0.63–0.98	**0.029**
	Fourth quintile	0.62	0.49–0.78	**<0.001**
	Fifth quintile	0.52	0.39–0.70	**<0.001**
Physical effort at work/main activity	Mostly sitting (Ref)			
	Mostly consists of walking/light effort	0.58	0.44–0.75	**<0.001**
	Mostly walks or does moderate physical exertion	0.62	0.52–0.73	**<0.001**
	Does mostly heavy, strenuous physical work	0.70	0.49–0.98	**0.037**
	Does not perform such activities or work of any kind	1.53	1.10–2.13	**0.011**
Alcohol consumption	High-risk (Ref)			
	Moderate alcohol user	0.59	0.43–0.82	**0.002**
	Non-drinker of alcohol	0.64	0.45–0.90	**0.011**
Fruit consumption	Every day (Ref)			
	Once or more a week	1.10	0.92–1.31	0.292
	Less than once a week	1.33	1.00–1.76	**0.047**
Vegetable consumption	Everyday (Ref)			
	Once or more a week	1.27	1.08–1.50	**0.005**
	Less than once a week	1.53	1.15–2.02	**0.003**
Fruit juice consumption	Everyday (Ref)			
	Once or more a week	1.27	1.01–1.61	**0.044**
	Less than once a week	1.30	1.01–1.67	**0.039**
Coffee consumption	3 or more times a day (Ref)			
	1–2 times a day	0.76	0.65–0.89	**0.001**
	Less than once a day	0.86	0.62–1.18	0.353
Sweetener for hot drinks	Natural sweetener (Ref)			
	Artificial sweetener	1.15	0.96–1.38	0.127
	No sweetener	1.00	0.82–1.21	0.972
Processed meat consumption	4–7 times a week (Ref)			
	1–3 times a week	1.21	1.03–1.41	**0.019**
	Less than once a week	1.53	1.21–1.93	**<0.001**
Fish consumption	4–7 times a week (Ref)			
	1–3 times a week	0.82	0.48–1.42	0.486
	Less than once a week	1.06	0.62–1.79	0.836
Dairy product consumption	4–7 times a week			
	1–3 times a week	1.05	0.88–1.24	0.608
	Less than once a week	1.11	0.88–1.41	0.376

Bold values indicate statistical significance (*p* < 0.05). Odds ratios (OR) are adjusted for variables in the ordinal regression model.

## Data Availability

The data analyzed in this study is subject to the following licenses/restrictions: The data presented in this study are available upon request from Hungarian Central Statistical Office who performed and supervised the data collection. Requests to access these datasets should be directed to Hungarian Central Statistical Office, www.ksh.hu/?lang=en (accessed on 25 May 2025).
